# (1*E*)-6-Meth­oxy-3,4-dihydro­naphthalen-1(2*H*)-one oxime

**DOI:** 10.1107/S1600536810034574

**Published:** 2010-09-04

**Authors:** Da-Cheng Jin, Feng-Yu Piao, Rong-Bi Han

**Affiliations:** a Testing Center, Yanbian University, Yanji 133000, People’s Republic of China; b Agricultural College of Yanbian University, Longjing 133400, People’s Republic of China; c Key Laboratory of Organism Functional Factors of the Changbai Mountain, Yanbian University, Ministry of Education, Yanji 133000, People’s Republic of China

## Abstract

In the crystal structure of the title compound, C_11_H_13_NO_2_, the mol­ecules are paired into centrosymmetric dimers *via* inter­molecular O—H⋯N hydrogen bonds.

## Related literature

For the biological activity of benzazepine derivatives, see: Wei *et al.* (2009 ▶). For details of the synthesis, see: Hester (1967 ▶).
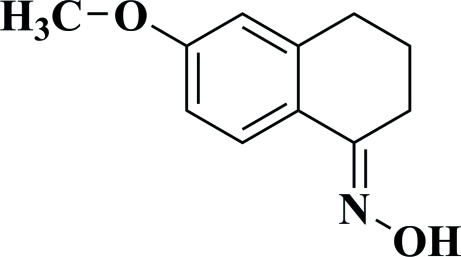


## Experimental

### 

#### Crystal data

C_11_H_13_NO_2_
*M* *_r_* = 191.22
Monoclinic, 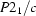

*a* = 8.185 (6) Å
*b* = 15.878 (10) Å
*c* = 8.053 (5) Å
β = 109.02 (3)°
*V* = 989.4 (11) Å^3^
*Z* = 4
Mo *K*α radiation
μ = 0.09 mm^−1^
*T* = 290 K
0.12 × 0.11 × 0.09 mm

#### Data collection

Rigaku R-AXIS RAPID diffractometer
Absorption correction: multi-scan (*ABSCOR*; Higashi, 1995 ▶) *T*_min_ = 0.989, *T*_max_ = 0.992
9568 measured reflections
2260 independent reflections
1724 reflections with *I* > 2σ(*I*)
*R*_int_ = 0.024

#### Refinement

*R*[*F* ^2^ > 2σ(*F* ^2^)] = 0.045
*wR*(*F* ^2^) = 0.140
*S* = 1.10
2260 reflections
129 parameters
H-atom parameters constrained
Δρ_max_ = 0.14 e Å^−3^
Δρ_min_ = −0.20 e Å^−3^

### 

Data collection: *RAPID-AUTO* (Rigaku, 1998 ▶); cell refinement: *RAPID-AUTO*; data reduction: *CrystalStructure* (Rigaku/MSC, 2002 ▶); program(s) used to solve structure: *SHELXS97* (Sheldrick, 2008 ▶); program(s) used to refine structure: *SHELXL97* (Sheldrick, 2008 ▶); molecular graphics: *PLATON* (Spek, 2009 ▶); software used to prepare material for publication: *SHELXL97*.

## Supplementary Material

Crystal structure: contains datablocks global, I. DOI: 10.1107/S1600536810034574/cv2749sup1.cif

Structure factors: contains datablocks I. DOI: 10.1107/S1600536810034574/cv2749Isup2.hkl

Additional supplementary materials:  crystallographic information; 3D view; checkCIF report

## Figures and Tables

**Table 1:** Hydrogen-bond geometry (Å, °)

*D*—H⋯*A*	*D*—H	H⋯*A*	*D*⋯*A*	*D*—H⋯*A*
O1—H1⋯N1^i^	0.82	2.09	2.805 (2)	146

Symmetry code: (i) 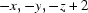.

**Fig. 1.**
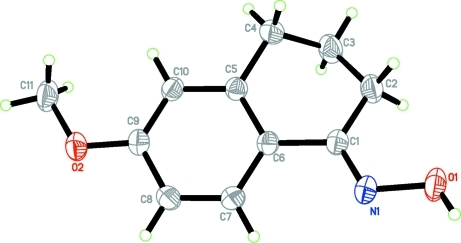
The molecular structure of the title compound showing the atomic numbering. Displacement ellipsoids of non-H atoms are drawn at the 30% probalility level.

## supplementary crystallographic information

### Comment

As a part of our study of 2,3,4,5-tetrahydro-7-methoxy-1*H*-2-benzazepin-1-one and its isomer, which exhibit anticonvulsant activities (Wei *et al.*, 2009), we report here the crystal structure of the title compound, which was used in our attempts to improve the selectivity of Backmann rearrangement.

In the title compound (Fig. 1) all bond lengths and angles are normal. Intermolecular O—H···N hydrogen bonds (Table 1) link molecules into centrosymmetric dimer. The cystal packing is further stablized by van der Waals forces.

### Experimental

The title compound was prepared according to the literature (Hester *et al.*, 1967). Colourless single crystals suitable for X-ray diffraction were cultured from a solution of 95% alcohol by slow evaporation at room temperature.

### Refinement

All H atoms were posioned geometrically and refined using a rding model, with C—H = 0.93–0.97 Å, O—H = 0.82 Å, and with *U*_iso_(H) = 1.2 or 1.5 *U*_eq_(C, O).

### Crystal data

**Table:**

C_11_H_13_NO_2_	*F*(000) = 408
*M**_r_* = 191.22	*D*_x_ = 1.284 Mg m^−^^3^
Monoclinic, *P*2_1_/*c*	Mo *K*α radiation, λ = 0.71073 Å
Hall symbol: -P 2ybc	Cell parameters from 6826 reflections
*a* = 8.185 (6) Å	θ = 3.1–27.5°
*b* = 15.878 (10) Å	µ = 0.09 mm^−^^1^
*c* = 8.053 (5) Å	*T* = 290 K
β = 109.02 (3)°	Block, colourless
*V* = 989.4 (11) Å^3^	0.12 × 0.11 × 0.09 mm
*Z* = 4	

### Data collection

**Table:**

Rigaku R-AXIS RAPID diffractometer	2260 independent reflections
Radiation source: fine-focus sealed tube	1724 reflections with *I* > 2σ(*I*)
graphite	*R*_int_ = 0.024
ω scans	θ_max_ = 27.5°, θ_min_ = 3.3°
Absorption correction: multi-scan (*ABSCOR*; Higashi, 1995)	*h* = −10→9
*T*_min_ = 0.989, *T*_max_ = 0.992	*k* = −20→20
9568 measured reflections	*l* = −10→10

### Refinement

**Table:**

Refinement on *F*^2^	Primary atom site location: structure-invariant direct methods
Least-squares matrix: full	Secondary atom site location: difference Fourier map
*R*[*F*^2^ > 2σ(*F*^2^)] = 0.045	Hydrogen site location: inferred from neighbouring sites
*wR*(*F*^2^) = 0.140	H-atom parameters constrained
*S* = 1.10	*w* = 1/[σ^2^(*F*_o_^2^) + (0.0809*P*)^2^ + 0.0477*P*] where *P* = (*F*_o_^2^ + 2*F*_c_^2^)/3
2260 reflections	(Δ/σ)_max_ = 0.022
129 parameters	Δρ_max_ = 0.14 e Å^−^^3^
0 restraints	Δρ_min_ = −0.20 e Å^−^^3^

### Special details

**Table:**

Geometry. All e.s.d.'s (except the e.s.d. in the dihedral angle between two l.s. planes) are estimated using the full covariance matrix. The cell e.s.d.'s are taken into account individually in the estimation of e.s.d.'s in distances, angles and torsion angles; correlations between e.s.d.'s in cell parameters are only used when they are defined by crystal symmetry. An approximate (isotropic) treatment of cell e.s.d.'s is used for estimating e.s.d.'s involving l.s. planes.
Refinement. Refinement of *F*^2^ against ALL reflections. The weighted *R*-factor *wR* and goodness of fit *S* are based on *F*^2^, conventional *R*-factors *R* are based on *F*, with *F* set to zero for negative *F*^2^. The threshold expression of *F*^2^ > σ(*F*^2^) is used only for calculating *R*-factors(gt) *etc*. and is not relevant to the choice of reflections for refinement. *R*-factors based on *F*^2^ are statistically about twice as large as those based on *F*, and *R*- factors based on ALL data will be even larger.

### Fractional atomic coordinates and isotropic or equivalent isotropic displacement parameters (Å^2^)

**Table:**

	*x*	*y*	*z*	*U*_iso_*/*U*_eq_	
C1	0.22438 (15)	0.07109 (8)	0.84267 (17)	0.0453 (3)	
C2	0.19968 (18)	0.05982 (10)	0.65136 (18)	0.0588 (4)	
H2A	0.0874	0.0819	0.5831	0.071*	
H2B	0.2007	0.0001	0.6260	0.071*	
C3	0.33762 (19)	0.10345 (11)	0.59483 (18)	0.0660 (4)	
H3A	0.3314	0.0837	0.4790	0.079*	
H3B	0.3160	0.1636	0.5874	0.079*	
C4	0.51630 (18)	0.08688 (11)	0.72171 (18)	0.0601 (4)	
H4A	0.5418	0.0272	0.7225	0.072*	
H4B	0.6009	0.1170	0.6836	0.072*	
C5	0.52944 (15)	0.11461 (7)	0.90409 (15)	0.0421 (3)	
C6	0.38514 (15)	0.10886 (7)	0.95932 (15)	0.0408 (3)	
C7	0.39938 (17)	0.13786 (8)	1.12760 (17)	0.0518 (3)	
H7	0.3033	0.1355	1.1650	0.062*	
C8	0.55144 (17)	0.16968 (9)	1.23853 (18)	0.0545 (4)	
H8	0.5583	0.1883	1.3501	0.065*	
C9	0.69544 (15)	0.17400 (8)	1.18325 (16)	0.0466 (3)	
C10	0.68406 (15)	0.14690 (8)	1.01691 (16)	0.0454 (3)	
H10	0.7803	0.1502	0.9799	0.055*	
C11	0.98486 (18)	0.22289 (12)	1.2455 (2)	0.0728 (5)	
H11A	1.0245	0.1709	1.2110	0.109*	
H11B	1.0762	0.2478	1.3397	0.109*	
H11C	0.9512	0.2608	1.1472	0.109*	
N1	0.11395 (13)	0.04621 (7)	0.91439 (15)	0.0541 (3)	
O1	−0.03579 (13)	0.01124 (8)	0.79076 (14)	0.0707 (4)	
H1	−0.0958	−0.0108	0.8427	0.106*	
O2	0.84142 (12)	0.20688 (7)	1.30220 (13)	0.0634 (3)	

### Atomic displacement parameters (Å^2^)

**Table:**

	*U*^11^	*U*^22^	*U*^33^	*U*^12^	*U*^13^	*U*^23^
C1	0.0398 (6)	0.0452 (6)	0.0489 (7)	−0.0035 (5)	0.0115 (5)	−0.0013 (5)
C2	0.0542 (8)	0.0699 (9)	0.0462 (7)	−0.0122 (7)	0.0077 (6)	−0.0058 (6)
C3	0.0646 (9)	0.0886 (11)	0.0403 (7)	−0.0151 (8)	0.0110 (6)	−0.0007 (7)
C4	0.0559 (8)	0.0813 (10)	0.0469 (8)	−0.0102 (7)	0.0219 (6)	−0.0115 (7)
C5	0.0415 (6)	0.0433 (6)	0.0406 (6)	−0.0005 (5)	0.0122 (5)	0.0015 (5)
C6	0.0397 (6)	0.0397 (6)	0.0420 (6)	−0.0028 (5)	0.0117 (5)	0.0004 (5)
C7	0.0469 (7)	0.0615 (8)	0.0516 (8)	−0.0096 (6)	0.0224 (6)	−0.0074 (6)
C8	0.0546 (7)	0.0654 (9)	0.0454 (7)	−0.0120 (6)	0.0190 (6)	−0.0117 (6)
C9	0.0419 (6)	0.0481 (7)	0.0453 (7)	−0.0054 (5)	0.0081 (5)	0.0001 (5)
C10	0.0374 (6)	0.0529 (7)	0.0460 (7)	−0.0019 (5)	0.0137 (5)	0.0019 (5)
C11	0.0445 (7)	0.0990 (13)	0.0673 (10)	−0.0210 (8)	0.0077 (7)	−0.0048 (9)
N1	0.0399 (6)	0.0618 (7)	0.0579 (7)	−0.0126 (5)	0.0124 (5)	−0.0070 (5)
O1	0.0464 (6)	0.0917 (8)	0.0675 (7)	−0.0275 (5)	0.0098 (5)	−0.0114 (6)
O2	0.0465 (5)	0.0848 (7)	0.0525 (6)	−0.0161 (5)	0.0072 (4)	−0.0123 (5)

### Geometric parameters (Å, °)

**Table:**

C1—N1	1.2833 (17)	C6—C7	1.3997 (19)
C1—C6	1.4724 (18)	C7—C8	1.3703 (19)
C1—C2	1.498 (2)	C7—H7	0.9300
C2—C3	1.516 (2)	C8—C9	1.3907 (19)
C2—H2A	0.9700	C8—H8	0.9300
C2—H2B	0.9700	C9—O2	1.3681 (16)
C3—C4	1.509 (2)	C9—C10	1.3808 (19)
C3—H3A	0.9700	C10—H10	0.9300
C3—H3B	0.9700	C11—O2	1.415 (2)
C4—C5	1.5032 (19)	C11—H11A	0.9600
C4—H4A	0.9700	C11—H11B	0.9600
C4—H4B	0.9700	C11—H11C	0.9600
C5—C10	1.3930 (19)	N1—O1	1.4164 (16)
C5—C6	1.3940 (18)	O1—H1	0.8200
			
N1—C1—C6	116.90 (12)	C5—C6—C7	118.39 (11)
N1—C1—C2	123.19 (12)	C5—C6—C1	119.81 (12)
C6—C1—C2	119.86 (11)	C7—C6—C1	121.79 (11)
C1—C2—C3	112.97 (11)	C8—C7—C6	121.59 (12)
C1—C2—H2A	109.0	C8—C7—H7	119.2
C3—C2—H2A	109.0	C6—C7—H7	119.2
C1—C2—H2B	109.0	C7—C8—C9	119.60 (13)
C3—C2—H2B	109.0	C7—C8—H8	120.2
H2A—C2—H2B	107.8	C9—C8—H8	120.2
C4—C3—C2	111.67 (13)	O2—C9—C10	124.44 (11)
C4—C3—H3A	109.3	O2—C9—C8	115.63 (12)
C2—C3—H3A	109.3	C10—C9—C8	119.93 (12)
C4—C3—H3B	109.3	C9—C10—C5	120.47 (11)
C2—C3—H3B	109.3	C9—C10—H10	119.8
H3A—C3—H3B	107.9	C5—C10—H10	119.8
C5—C4—C3	110.81 (12)	O2—C11—H11A	109.5
C5—C4—H4A	109.5	O2—C11—H11B	109.5
C3—C4—H4A	109.5	H11A—C11—H11B	109.5
C5—C4—H4B	109.5	O2—C11—H11C	109.5
C3—C4—H4B	109.5	H11A—C11—H11C	109.5
H4A—C4—H4B	108.1	H11B—C11—H11C	109.5
C10—C5—C6	120.00 (12)	C1—N1—O1	112.30 (12)
C10—C5—C4	120.37 (11)	N1—O1—H1	109.5
C6—C5—C4	119.62 (11)	C9—O2—C11	118.02 (12)

### Hydrogen-bond geometry (Å, °)

**Table:**

*D*—H···*A*	*D*—H	H···*A*	*D*···*A*	*D*—H···*A*
O1—H1···N1^i^	0.82	2.09	2.805 (2)	146

Symmetry codes: (i) −*x*, −*y*, −*z*+2.
